# Modern Paediatric Radiology: Meeting the Challenges in CT and MRI

**DOI:** 10.31729/jnma.7539

**Published:** 2022-07-31

**Authors:** Pradeep Raj Regmi, Isha Amatya, Sharma Paudel, Prakash Kayastha

**Affiliations:** 1 Department of Radiology and Imaging, Tribhuvan University Teaching Hospital, Maharajgunj, Kathmandu, Nepal; 2 Health Research Section, Nepal Health Research Council, Ramshahpath, Kathmandu, Nepal

**Keywords:** *computed X-ray tomography*, *magnetic resonance imaging*, *pediatrics*, *radiation exposure*, *X-ray*

## Abstract

Radiology plays a very important part in the diagnosis, treatment, and follow-up of children. Computed tomography and magnetic resonance imaging are the two most crucial developments in the modern era. However, the two modalities have their challenges to overcome. Radiation dose is the most unwanted side effect of computed tomography scans while longer scan time along with sedation is a major disadvantage in children during magnetic resonance imaging. Paediatric-specific protocol selection and limiting the exposure to the area of interest aid in reducing the dose during computed tomography scans. Faster scan protocols and sequences can result in imaging without sedation in magnetic resonance imaging. Considering the radiation exposure, "as low as reasonably achievable" principle should be followed strictly in the paediatric population. In this article, possible ways for minimising the radiation dose in computed tomography, as well as effective, short, and sedation-free magnetic resonance imaging, are discussed.

## INTRODUCTION

Computed tomography (CT) and magnetic resonance imaging (MRI) are the preferred modalities for clinicians all over the world. However, there are challenges these two modalities have to overcome to become as efficient in children as compared to adults. Radiation protection is especially very important in children undergoing CT scans. Since the child has a higher life expectancy than the adult population, the chance of having the manifestation of harmful side effects of radiation is higher.^1^ The tissue with more mitosis and a large proportion of hematopoietic tissue is at higher risk from the radiation exposure which is expected in the growing population. In addition, a child's body has more water content so that more radiation is dispersed and an even higher dose is required to penetrate the same thickness as an adult. Therefore, the risk of radiation is 10-11 times higher in the paediatric population than in the adult population.^1,2^

## MINIMISING RADIATION DOSE IN CT

The use of CT has increased drastically over the last decades. In the United States, nearly 43% of children underwent evaluation using radiation within 3 years, and nearly 8% of the study population had a CT over the same period.^3,4^ Thus, with the great improvement in the diagnostic capabilities, comes the great risk of radiation. The estimated lifetime risk of death from cancer that is attributable to a CT scan is calculated by summing the estimated organ-specific cancer risks.^5^ The risks are highly dependent on age because both the doses and the risks per unit dose are age-dependent. Even though doses are higher for head scans, the risks are higher for abdominal scans because the digestive organs are more sensitive than the brain to radiation-induced cancer.^5^ These risk estimates are based on the organ doses which were derived for average CT machine settings. This risk is inversely related to the age of radiation exposure. Therefore, the main challenges in modern CT technology are to shorten the scan time and reduce the radiation dose as low as possible. Some possible ways to achieve this are highlighted below:

Alternative modalities to give accurate diagnostic results other than CT could be considered. Strong consideration should be given to ultrasonography and MRI to minimise the risk of radiation in children.

Paediatric-specific protocol selection and limiting the CT protocols to the area of interest aid in reducing the dose. For example, for CT urography split bolus technique can be used instead of multiphasic scans for evaluation in urology except in vascular pathology or suspicious tumour cases. This could reduce as much as 50% of the dose in comparison to multiphasic scans.^6^ The split bolus technique for paediatric oncology patients showed the lowest range of radiation dose compared to chest or abdomen CT of the same age with non-compromised diagnostic efficacy.^7^ Using as low voltage as possible in the follow-up cases like ventriculoperitoneal shunts can lower the radiation dose.^8^

Many a time, parents are more anxious than the child. So, a proper explanation of the procedure to the parents is important to gain confidence. The confidence of the child can be gained in various ways like a simulation of the imaging process before real imaging, wall paintings, or some video clips inside the imaging room. Performing the imaging process without sedation is a bit challenging yet risk-free step which can be done by creating a favourable environment. Proper positioning, beam centring, collimation and shielding can reduce the radiation dose in children and also reduces the repeat examination due to artefacts or inadequate examinations.

Automatic current modulation is currently available in modern scanners. Tube current (mA) combined with gantry rotation time expressed as milliamperes seconds (mAs) has a linear relationship with the radiation dose. However, it should be lowered with caution since the decrease in mAs can increase the noise and decrease spatial resolution. The voltage has a non-linear relationship with the radiation dose. For example, an increase in kilovoltage peak (kVp) from 80 to 120 can increase the CT dose index volume four folds. Lowering the X-ray tube potential is among the most effective means of reducing the radiation dose associated with CT in children. So, mAs and kVp should be chosen cautiously to maximize the image contrast and reduce noise, and radiation dose kept as low as reasonably achievable.^8^

Dual-energy CT (DECT) can reduce the radiation dose in children through virtual non-enhancing imaging. It can quantify the iodine and non-iodine components of a tissue voxel, and the relative contribution of iodine to the attenuation of each voxel on a contrast-enhanced examination can be subtracted, thus simulating a non-enhanced image. Low virtual mono-energetic images (such as those at 40 keV) that approach the K-edge of iodine (33.2 keV) increase the conspicuity of intravenous iodinated contrast material, which can enable the use of a lower volume of contrast material and a lower injection rate.^9^

CT machines with large detector rows and fast scanning capabilities enable simultaneous use of both rapid ultra-high pitch scanning and a low-kilovolt setting (70-80 kV), lessening the need for sedation in children thereby reducing effective radiation dose without degrading the image quality.^8^

Adaptive dynamic collimation and dose notification alarms set within the scanners can effectively lower the dose due to overhanging and inadvertent overdose respectively.

Adaptive Statistical Iterative Reconstruction (ASIR) has also reduced the image noise through the computer software or can allow noise up to a certain limit so that image quality is not degraded. As much as 39% of the radiation dose in CT thorax and 29% in CT abdomen is reduced in children with this technique.^10^

## REDUCING SCAN TIME IN MRI

The major challenge in the present paediatric MRI in the current scenario is to shorten the duration of imaging and perform the scan without sedation. The term fast in this article is used to denote the shorter scan time in MRI. It is extremely difficult without specific paediatric protocols and proper teamwork. Sedation procedures have their disadvantages and risks of complications to the sick child. The ways for effective, short, and sedation-free MRI scan are hereby mentioned below:

Similar to CT, pre-imaging sessions with audiovisual, mock MRI can aid in preparation and gaining the confidence of the child and the family for efficient scans to make the child calm thereby performing the procedure sedation free. Especially for neonatal MRI, the first-line method is the feed and swaddle technique which coordinated the scan time with sleeping time.

The use of paediatric-specific coils and the MRI-compatible monitoring sets especially needed for neonatal patients can reduce the signal-to-noise ratio and diagnostic difficulties.

Checking the images after each scan can be a great help for children to reduce the scan time so that sequences that yield the most information should be done earliest.

Faster brain MRI protocols (especially for follow-up cases of ventriculo-peritoneal shunt, congenital anomalies, or traumatic brain injury) can avoid the need for anaesthesia or sedation and avoid the radiation of CT. It lies on the principle to eliminate the sequence providing minimal information about the probable diagnosis. For example, in a child with known shunted hydrocephalus and clinical concern for shunt dysfunction, a rapid nonsedated MRI with only Single Shot Fast Spin ECHO (SSFSE) images is sufficient to compare the ventricular size to the prior scan.^11,12^

Respiratory gating techniques are helpful in the paediatric population to lessen respiratory artefacts. Scheduling the procedure during nap time can also reduce motion artefacts. Sequences less sensitive to motion artefacts like Periodically Rotated Overlapping Parallel Lines with Enhanced Reconstruction (PROPELLER) can help. 3D acquisitions can be faster than 2D in many scenarios.^13^

Parallel imaging, simultaneous multisection imaging, radial k-space acquisition, compressed sensing MRI reconstruction, and automated protocol selection software effectively reduces the time of MRI acquisition in children.^14^

## WAY FORWARD

CT and MRI are valuable modalities for paediatric patients. Considering the more radiation-related risks in children compared to adults, as low as reasonably achievable (ALARA) principle should be followed strictly in the paediatric population. To minimise the use of sedation and its unwanted complications while still addressing the clinical indication, practising to cut short the scan time i.e Focused Assessment with Sonography for Trauma (FAST) scan in MRI is equally important in today's scenario of paediatric radiology. Radiologists and Radio-technicians must be aware of and address these issues in current clinical practice in this modern era of diagnostic medicine.
